# Metallic conduction and large electron-phonon-impurity interference effect in single TiSi nanowires

**DOI:** 10.1186/1556-276X-7-500

**Published:** 2012-09-05

**Authors:** Wei-Che Hsu, Chao-Chun Chen, Yong-Han Lin, Huang-Kai Lin, Hsin-Tien Chiu, Juhn-Jong Lin

**Affiliations:** 1 Institute of Physics, National Chiao Tung University, Hsinchu, 30010, Taiwan; 2 Department of Applied Chemistry, National Chiao Tung University, Hsinchu, 30010, Taiwan; 3 Department of Electrophysics, National Chiao Tung University, Hsinchu, 30010, Taiwan

**Keywords:** Chemical vapor deposition reaction, TiSi nanowire, Silicide, Electron-phonon scattering, Electron-phonon-impurity interference, Focused-ion-beam-induced deposition

## Abstract

We report on the first electrical characterizations of single-crystalline TiSi nanowires (NWs) synthesized by chemical vapor deposition reactions. By utilizing the focused-ion-beam-induced deposition technique, we have delicately made four-probe contacts onto individual NWs. The NW resistivities have been measured between 2 and 300 K, which reveal overall metallic conduction with small residual resistivity ratios in the NWs. Surprisingly, we find that the effect due to the interference processes between the elastic electron scattering and the electron-phonon scattering largely dominates over the usual Boltzmann transport even at room temperature. Such prominent electron-phonon-impurity interference effect is ascribed to the presence of large amounts of disorder and high Debye temperatures in TiSi NWs.

## Background

Physical properties of transition metal silicides are intensively investigated for their potential usefulness in many device applications. Among them, titanium silicides constitute a valuable material family that is widely utilized as gate electrodes and interconnects in ultra-large-scale integrated circuits, owing to their relatively low electrical resistivities and good thermal and chemical stability that is highly compatible with present-day silicon processes [1]. Among all the six phases of titanium silicides reported to date, titanium monosilicide (TiSi) [2] has the highest mechanical hardness which, combined with its low resistivity [3] (≈ 60 *μΩ* cm in bulk form at 300 K), can be of interest for the possible engineering of micro- and nano-electromechanical systems [4]. However, as compared to other titanium silicides, TiSi has not been much studied either in bulk form or at the nanoscale level due to the difficulties of either preparing quantities sufficient for bulk characterizations [3] or preparing single-phased nanostructures [5].

Recently, some of us have successfully synthesized single-crystalline TiSi nanowires (NWs) [5]. The material belongs to one of the eight transition metal silicide NWs currently known [6]. In this work, we aim to study the intrinsic electrical transport properties of this nanoscale material by measuring four-probe individual NW samples in a wide temperature range of 2 to 300 K. We demonstrate that these NWs are indeed metallic, revealing decreasing resistivity with reducing temperature. Furthermore, we observe a large electron-phonon-impurity (EPI) interference effect, which is theoretically predicted to exist in disordered conductors [7]. Surprisingly, we find that this EPI interference effect strongly dominates over the usual Boltzmann transport even at temperatures as high as room temperature. This unique property renders the TiSi NWs useful for the investigations of the interplay among a variety of electron-scattering processes at the nanoscale level. Previously, the EPI interference effect has been found to be important in normal metals only at considerably lower temperatures [8-12].

## Methods

Our TiSi NWs were synthesized via a low-pressure chemical vapor deposition process using TiCl_4_ and titanium powder as precursors; no templates or catalysts were needed [5]. The growth mechanism and detailed structure and composition characterizations by high-resolution transmission electron microscopy (HRTEM), X-ray diffraction (XRD), and other techniques have been reported by Lin et al. in [5]. Since these NWs can be readily coated by a thick (approximately 10 nm) oxide layer over the surfaces [5,13], we choose the ‘invasive’ focused-ion-beam-induced deposition (FIBID) technique to fabricate the electrical contacts onto individual NWs. It should be stressed that the existence of the thick oxide layers prevented us from making good ohmic contacts when employing the electron-beam lithographic technique. After transferring the NWs onto the silicon substrate capped with a ≈ 500-nm thick SiO_2_ layer, the positions of individual NWs were first located by the scanning electron microscopy (SEM) capability of the FIB system (Model FEI Nova 200, FEI Co., Hillsboro, OR, USA). Platinum electrodes were then deposited onto the NWs using a 30-KeV, 10-pA Ga^+^ FIB. The Pt leads connected the individual NWs to the microelectrodes which were photolithographically pre-patterned on the SiO_2_/Si substrate. The substrate was thermally anchored to the sample holder mounted with a calibrated silicon diode thermometer on a standard ^4^He cryostat. A standard four-probe current-reversal method was applied for resistance measurements using a Keithley K-220 current source and a K-182 nanovoltmeter (Keithley Instruments Inc., Cleveland, OH, USA).

**Figure 1:**
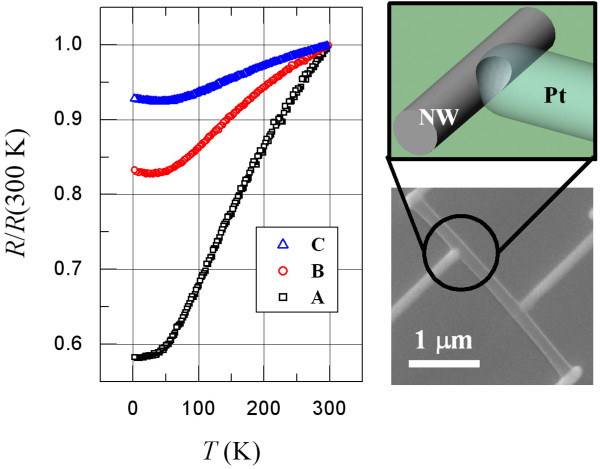
***R/R *(300 K) versus *T *for three TiSi NW samples.** The right panels show the schematic depicting the Pt-electrode/TiSi-NW contact made by the FIBID technique and the SEM image of the NW sample.

Since the NWs are readily coated with a thick oxide layer, as mentioned, we apply the FIBID technique in a delicate manner to unambiguously obtain the *intrinsic* electrical transport properties of the NWs. Instead of being laid across the entire NW diameter, the deposition of the Pt (especially the two *voltages*) electrodes were carefully brought only to the edge of the NW, which removed the outer oxide layer of the NW by invasive deposition and allowed the deposited Pt leads to be in direct contact with the fresh interior of the NW (see the schematic in the right panel of Figure 1). This approach prevents the TiSi NW from being broken into disconnected regimes as the bombardment of high-energy Ga^+^ ions during the FIBID could seriously damage and/or mill away the entire NW segment under the Pt electrodes, in which case a nominal four-probe measurement could become ineffective and give a two-probe result that includes the resistances of the Pt segments in the contact regions of the two voltage electrodes. (Due to the presence of Ga/C contents in the electrodes, the as-deposited ‘Pt’ electrodes are typically slightly insulating. In this work, the Pt electrodes consist of approximately 70% C, 20% Pt, and 10% Ga and have resistivity *ρ*_el_(300 K)≈ 3,000 *μΩ* cm and resistivity ratio *ρ*_el_(4 K)/*ρ*_el_(300 K)≈ 1.7, which are comparable to those values reported in the literature [14-16]. Typically, in all cases, our Pt electrodes have resistances of approximately a few ten kilohms, and the contact resistances are estimated to be only a few hundred ohms.) The normalized resistance *R*/*R*(300 K) versus temperature for three single NW samples fabricated this way is shown in Figure 1. Obviously, we obtain overall metallic behavior, i.e., the resistance decreases with decreasing temperature from 300 K down to low temperature. It should be mentioned that this technical precaution has often been overlooked, and great care should be taken when applying this invasive contact technique to electrical characterizations of nanoscale conductors.

## Results and discussion

Table 1 indicates that our measured *ρ*(300 K) ≈ 310 to 350 *μΩ* cm values are a few times higher than that (≈ 60 *μΩ* cm) reported for bulk TiSi [3]. Our measured low residual resistivity ratios *ρ*(300 K)/*ρ*_0_<2 strongly suggest the presence of large amounts of defects (e.g., point defects) in the NWs, where *ρ*_0_ is the residual resistivity due to elastic electron scattering off defects. This observation is in sharp contrast to the conclusion drawn from HRTEM and XRD studies [5], where structure characterizations indicated good crystalline structures without noticeable lattice defects. Indeed, the electrical transport measurement is one of the most sensitive techniques for probing defects in single nanoscale structures [17-19].

**Table 1:** Values of relevant parameters for three TiSi NW samples

**Sample**	**Diameter**	***ρ*(300 K)**	**_*ρ*0_**	***ρ*(300 K)/_*ρ*0_**	**_*β*BG_**	**_*θ*D_**	**_*β*int_**
	**(nm)**	**(*μΩ* cm)**	**(*μΩ* cm)**		**(*μΩ* cm K^-1^)**	**(K)**	**(K^-2^)**
A	190	310	180	1.72	0.87	520	1.7 ×1^0−5^
B	150	350	290	1.21	0.13	650	5.5 ×1^0−6^
C	100	350	330	1.06	0.10	680	1.8 ×1^0−6^

Note that the thickness of the oxide layer (approximately 10 nm) is subtracted in the estimated NW diameters.

In the standard electrical transport model, the temperature behavior of resistivity *ρ* in metals is described by Matthiessen’s rule: *ρ*(*T*)=*ρ*_0_ + *ρ*_BG_(*T*), where the Bloch-Grüneisen term, *ρ*_BG_(*T*), due to electron-phonon scattering in an impure metal is given by [20,21]

(1) $$\rho_{\mathrm{BG}}(T)=\beta_{\mathrm{BG}}T{\left(\frac{T}{\theta_{D}}\right)}^{4}\overset{\theta_{D}/T}{\underset{0}{\int }}\frac{x^{5}\mathrm{dx}}{\left(e^{x}-1)(1-e^{-x}\right)}\;,$$

where *β*_BG_ is a material-dependent electron-phonon coupling parameter, and *θ*_D_is the Debye temperature. Previously, the applicability of using the Bloch-Grüneisen theorem for electron-phonon resistivity in metallic NWs of diameter ≥ 15 nm has been established [22]. In practice, however, deviations from Matthiessen’s rule are often seen in metals that contain disorder [23]. Recently, Reizer and Sergeev [7] have proposed that such deviations can be theoretically explained by taking into account the various interference processes generated between the elastic electron scattering and the electron-phonon scattering. They predicted that this new EPI interference mechanism causes a new resistivity contribution [7]

(2) $$\;\rho_{\mathrm{int}}(T)\;=\;\beta_{\mathrm{int}}T^{2}\rho_{0}\left(\frac{6}{\Pi^{2}}\right)\overset{\theta_{D}/T}{\underset{0}{\int }}\left[\frac{x^{2}e^{x}}{{\left(e^{x}\;-\;1\right)}^{2}}\;-\;\frac{x}{e^{x}\;-\;1}\right]\mathrm{dx}\;,$$

where *β*_int_ is a material-dependent electron-phonon coupling parameter. Thus, for a disordered metal, the total resistivity is

(3) $$\rho (T)=\rho_{0}+\rho_{\mathrm{BG}}(T)+\rho_{\mathrm{int}}(T)\;.$$

(The quantum interference weak localization and electron-electron interaction effects [24] that could arise at lower temperatures are ignored here. They contribute negligibly to the measured resistivity in the temperature range of interest in the present work.)

**Figure 2:**
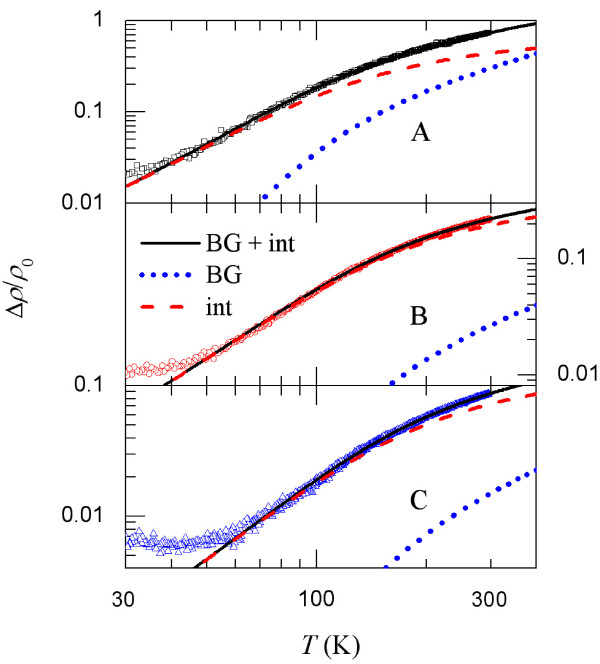
**Variations of **Δ*ρ/ρ*_0 _**with temperature for three TiSi NW samples (A, B, and C).** The solid curves are least-squares fits to Equation 3. Individual contributions of Equations 1 and 2 are also plotted, as indicated.

We have least-squares fitted our measured *ρ*(*T*) in the NWs to Equation 3, with *ρ*_0_, *β*_BG_, *θ*_D_, and *β*_int_as adjusting parameters. Figure 2 shows the normalized resistivity, *Δρ*/*ρ*_0_=(*ρ*−*ρ*_0_)/*ρ*_0_, versus temperature for the three NW samples. Good agreement between theory and experiment is found for every NW. Our fitted *θ*_D_values (≈ 520 to 680 K) are relatively high (Table 1) compared with, e.g., those in noble metals. Our fitted values of $\beta_{i\mathrm{nt}}∼10^{-6}$−1^0−5^K^−2^ are in similar orders of magnitude with those previously obtained in normal metals, such as Au [8], Al [9], and AuPd [11]. What is more interesting is that previous studies of a variety of metals have found that the EPI interference effect was important only at considerably low temperatures [8-12]. In sharp contrast, we find that in our TiSi NWs, *ρ*_int_(*T*) can largely dominate over *ρ*_BG_(*T*) even up to temperatures as high as room temperature. Figure 2 clearly illustrates that *ρ*_int_(*T*) is about one order of magnitude larger than *ρ*_BG_(*T*) in samples B and C at 300 K.

Since the EPI interference effect is governed by the various interference processes generated between the elastic electron scattering and the electron-phonon scattering, our observation may be understood as follows. For a given material characterized by the same *β*_BG_, *θ*_D_, and *β*_int_ values, when the amount of disorder (*ρ*_0_) contained in the sample increases, the number of electronic waves generated from the elastic scattering off defects increases correspondingly. This can consequently enhance the strength of the EPI interference effect, giving rise to an increasingly pronounced *ρ*_int_(*T*), as dictated by Equation 2. Furthermore, a metal with a high *θ*_D_value means that the number of phonons that can participate in the electron-phonon scattering is relatively large. (Note that the upper limits of the integrals in Equations 1 and 2 are given by *θ*_D_.) This can also lead to an enhanced EPI interference effect. As both the *ρ*_0_ and *θ*_D_ values in our TiSi NWs are higher than those in normal metals, a significant contribution due to such an effect hence can result.

**Figure 3:**
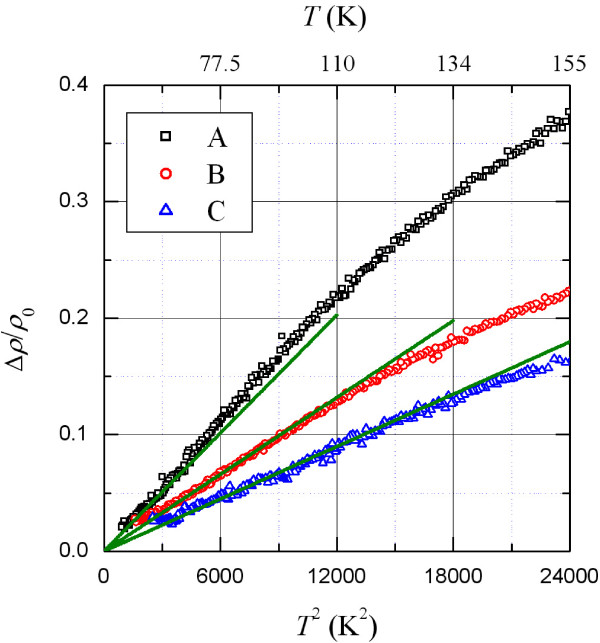
*Δρ/ ρ_0_***versus ***T*^2 ^**for three TiSi NW samples.** The straight lines are the predictions of the EPI interference effect in the limiting case: $\rho_{\text{int}}(T)≃\beta_{\text{int}}T^{2}\rho_{0}$. For clarity, the data for sample B (C) have been offset by multiplying a factor of 2 (4).

The importance of the EPI interference effect can also be directly checked by plotting *Δρ*/*ρ_0_*versus *T*^2^. Theoretically, the integral in Equation 2 approaches *Π*^2^/6 at $T≲0.1\theta_{D}$, and thus, Equation 2 reduces to a simple power-law form: $\rho_{\mathrm{int}}(T)≃\beta_{\mathrm{int}}T^{2}\rho_{0}$. This *T*^2^temperature characteristic would extend to higher temperatures in metals that possess higher *θ*_D_ values and contain larger amounts of disorder (*ρ*_0_), as mentioned previously. Figure 3 clearly demonstrates that this *T*^2^ law prevails in a wide temperature range of approximately 50 to 140 K in our most disordered sample, C. In sample B, the *T*^2^ law is seen between approximately 40 and 120 K.

Inspection of Table 1 indicates that our extracted *β*_BG_,*θ*_D_, and *β*_int_ parameters vary among samples. Such variations cannot be explained in terms of current theoretical concepts. According to the current theoretical understanding, the two electron-phonon coupling parameters *β*_BG_ and *β*_int_ are expected to be independent of disorder for a given material in the weakly disordered regime [7]. On the other hand, whether the value of *θ*_D_ should vary with disorder (or sound velocity which could be disorder dependent) is less clear [25]. These issues deserve further theoretical and experimental investigations.

## Conclusions

In summary, we have measured the temperature-dependent resistivity of single TiSi NWs between 2 and 300 K. We demonstrated that as-grown TiSi NWs are metallic. Furthermore, we found a large electron-phonon-impurity interference effect, which strongly dominates the total resistivity up to temperatures as high as room temperature. This is ascribed to originating from large Debye temperatures and high levels of disorder (e.g., point defects) in as-grown TiSi NWs. Our observations suggest that TiSi NWs can serve as a useful system for studying the rich electron scattering processes at the nanoscale level.

## Competing interests

The authors declare that they have no competing interests.

## Authors’ contributions

WCH conducted the electrical measurements. CCC realized the FIBID contacts. They both participated in the experiment design. YHL analyzed the results and wrote the manuscript. HKL and HTC carried out the synthesis and structure and composition characterizations of the NWs. JJL coordinated and supervised the overall study and helped draft the manuscript. All authors read and approved the final manuscript.
